# Interviews of living kidney donors to assess donation-related concerns and information-gathering practices

**DOI:** 10.1186/s12882-018-0935-0

**Published:** 2018-06-08

**Authors:** Jessica M. Ruck, Sarah E. Van Pilsum Rasmussen, Macey L. Henderson, Allan B. Massie, Dorry L. Segev

**Affiliations:** 1 Department of Surgery, Johns Hopkins University School of Medicine, 720 Rutland Ave, Ross 34, Baltimore, MD 21205 USA; 2 Department of Epidemiology, Johns Hopkins School of Public Health, 720 Rutland Ave, Ross 34, Baltimore, MD 21205 USA

**Keywords:** Living kidney donors, Concerns, Knowledge, Education

## Abstract

**Background:**

Efforts are underway to improve living kidney donor (LKD) education, but current LKD concerns and information-gathering preferences have not been ascertained to inform evidence-based resource development. As a result, prior studies have found that donors desire information that is not included in current informed consent and/or educational materials.

**Methods:**

We conducted semi-structured interviews with 50 LKDs who donated at our center to assess (1) concerns about donation that they either had personally before or after donation or heard from family members or friends, (2) information that they had desired before donation, and (3) where they sought information about donation. We used thematic analysis of verbatim interview transcriptions to identify donation-related concerns. We compared the demographic characteristics of participants reporting specific concerns using Fisher’s exact test.

**Results:**

We identified 19 unique concerns that participants had or heard about living kidney donation. 20% of participants reported having had no pre-donation concerns; 38% reported no post-donation concerns. The most common concern pre-donation was future kidney failure (22%), post-donation was the recovery process (24%), and from family was endangering their family unit (16%). 44% of participants reported being less concerned than family. 26% of participants wished they had had additional information prior to donating, including practical advice for recovery (10%) and information about specific complications (14%). Caucasian participants were more likely to hear at least one concern from family (76% vs. 33%, *p* = 0.02). The most commonly consulted educational resources were health care providers (100%) and websites (79% of donors since 2000). 26% of participants had had contact with other donors; an additional 20% desired contact with other LKDs.

**Conclusions:**

Potential donors not only have personal donation-related concerns but frequently hear donation-related concerns from family members and friends. Current gaps in donor education include an absence of practical, peer-to-peer advice about donation from other prior donors and materials directed and potential donors’ family members and friends. These findings can inform the development of new educational practices and resources targeted not only at LKDs but at their social networks.

## Background

Within the transplant community, knowledge of the risks associated with living kidney donation has increased as living kidney donors are more rigorously followed and studied. These risks include perioperative complications [1], financial costs [2, 3], hypertension [4–6], kidney disease and failure [7–11], pregnancy complications [12, 13], and death [4]. However, this information does not always reach the general public or, most importantly, potential donors. Studies have shown that the general public has unrealistic concerns regarding donation [14, 15] and that potential donors are unaware of what donation requires [16].

In order to educate donors and empower them to make informed decisions about donation, we must improve our approaches to donor education. While national policies require centers to inform potential donors about specific donation-related risks, comprehensive donor education must also address other concerns and possible misconceptions of potential living kidney donors. While several studies have examined concerns and information-gathering practices of donors [16–18], these predate many modern studies of donation-related risks [1, 4, 5, 7, 9–13, 19] as well as widespread use of the internet [20]. Given these changes in knowledge about donation-related risks and methods of information delivery in the last decade, it is difficult to optimize donor education without current information about donors’ concerns or how they seek information about living donation.

This study presents a qualitative and quantitative analysis of the concerns, misconceptions, and information-gathering behaviors of living kidney donors in the modern, internet-based era in the United States.

## Methods

### Study population

We recruited participants from living kidney donors enrolled in a longitudinal follow-up study at our center (Wellness and Health Outcomes of Live Donors Study) who donated at our center, consented to be contacted for future studies, and provided a telephone number. We interviewed a convenience sample of the first 50 donors to consent to the study (3 donors who were contacted declined). This study was approved by the Johns Hopkins Institutional Review Board, IRB00098726.

All interviews were conducted over a 3-day period. We called potential participants in batches of 50. After completing a batch of 50, potential participants who did not answer the phone were called a second time. Individuals who had answered and requested a call back at a different time were called at that alternate time. We continued to call potential participants until 50 interviews had been conducted. As a result, some individuals agreed to be interviewed at a later time but we reached out pre-determined sample size before their suggested call-back time.

A convenience sample of 50 participants was chosen to ensure both thematic saturation [21] and power for basic quantitative analysis, with the goal of exploration and prioritization of themes rather than quantifiable generalizability. Thematic saturation was reached after approximately 20 interviews, suggesting that major themes were likely captured. We compared the demographic characteristics of participants with those of donors who were called but did not complete the survey to assess potential participation bias.

### Current donor education practices

Potential living kidney donors who contact our center have several educational experiences to ensure they understand the risks, benefits, and process of living kidney donation. First, all living donors have a phone call with one of the transplant nurse coordinators. During this phone call, the nurse coordinator will go through a questionnaire, consent forms, and a handbook on living donation. All of the information covered during the phone call is sent via mail to the potential donor, along with a booklet that contains additional information about donation and a description of the donor pathway. The nurse coordinator then follows up with the donor at each step in the evaluation process and answers any questions that arise. If they donor is selected for the full evaluation day, during which the donor meets with the transplant surgeon, a nephrologist, and a social worker, the donor is retold all information during that evaluation visit.

### Interview script design

The first portion of the semi-structured interviews used open-ended questions to elicit concerns that donors recalled having prior to donation, after donation, or hearing from family members and/or friends. Of note, when we report concerns of family and friends, these concerns were heard second-hand from the donors and not directly from their family members or friends. Donors were also asked what they wished they had known prior to donating, as well as what else they would want us to know about any donation-related concerns they had; these questions sought to capture any concerns not previously elicited. The second portion of the interview involved asking the donors how often they used specific informational resources (e.g. website, books, scientific journals, healthcare providers) and how helpful each resource was on a scale of 0–10 (0 = Not helpful at all, 10 = Incredibly helpful). Participants were then asked to rate a list of qualities that these educational resources might have (e.g. from a reputable source, easy to read and understand, recommended by a friend) on a scale of 0–10 (0 = Not important at all, 10 = Extremely important). The interviews were concluded with an open-ended question to capture anything else the donors wanted us to know about how they obtained information about living kidney donation.

### Patient interviews

A single researcher (JR) with experience interviewing living kidney donors conducted all interviews. Participants were read a script stating that the study goals were to collect a list of concerns participants had or heard about living kidney donation, as well as to learn how participants obtained information about donation. Participants were read an oral consent form.

Interviews included twelve structured-response questions, seven free-response questions, and two structured-response follow-up questions that asked them to rank items on a scale of 0–10. Structured-response questions were used to assess the frequency with which informational resources were used and how helpful they were perceived to be. Structured-response questions were also used to measure the importance of various characteristics of an informational resource (e.g. coming from a reputable source or being recommended by a friend). Free-response questions were used to elicit concerns that participants had before or after donation, concerns expressed by family or friends, and information they wished they had had before donation. At the end of each section, participants were asked if they had any additional comments about donation-related concerns or information-gathering.

All interviews were recorded to optimize accuracy of subsequent content analysis and transcribed verbatim by the same researcher who had conducted the interviews (JR). Additionally, major themes were noted during interviews in field notes to assist in later analysis.

### Qualitative analysis

Responses to open-ended questions were independently coded by two members of the study team (JR and SVPR) using NVivo 11 Plus. Discrepancies in coding were reviewed and reconciled by the two coders. Themes from field notes were supplemented with themes derived from the transcribed data during coding. Average interview length, including administration of the questionnaire but not the telephone script or consent process, was approximately 15 min. We performed thematic saturation analysis by reviewing interviews chronologically and coding the first appearance of each theme. We found that 81% of the identified themes were revealed in the first 10 interviews, and only one new theme was uncovered in the last 20 interviews, suggesting that thematic saturation had been reached.

### Quantitative analysis

After themes around concerns were identified, each patient was coded as having each particular concern or not. Participants were separated into those who reported any concerns and those who did not report any concerns [1] pre-donation, [2] post-donation, or [3] from family. The percentage of participants reporting any concerns was compared across these three categories to test for associations with demographic characteristics (e.g. sex, race, employment status, and marital status) using Fisher’s exact test. We also compared the proportion of participants who reported personally having a concern (pre-donation or post-donation) to the proportion who reported hearing a concern from family or friends. All quantitative analyses were performed using Stata 14.1/MP for Windows (College Station, Texas).

## Results

### Study population

A convenience sample of 50 living kidney donors was interviewed. A total of 181 living kidney donors were called during recruitment; 10 (5.5%) could not be reached due to an incorrect or out-of-service phone number, 100 (55.2%) did not answer the phone, 3 (1.7%) declined to participate, and 18 (9.9%) asked to be recontacted later but were not reached before recruitment was completed. There were no significant differences between the demographic characteristics of participants and donors who were called but who did not complete the interview (Table 1).

**Table 1:** Characteristics of participants (*N* = 50) and contacted non-participants

Characteristic	% of Participants	% of Donors Who Were Contacted but Did Not Participate^a^	p
Sex, female	62%	62%	0.4
Race, Caucasian	82%	83%	0.8
Employed^b^	72%	82%	0.6
Married^b^	68%	71%	0.8
Age at donation, median (IQR)	46.5 (36, 55.5)	45 (36, 55)	0.8
Years since donation, median (IQR)	9 (6,14)	10 (5,15)	0.4

^a^Living kidney donors who were called but who did not answer their phone, declined the study, had an incorrect number listed in our database, or wished to participate but were not available prior to completing enrollment were included here as “donors who were contacted but did not participate”

^b^Employment and marital status at time of survey 1 administration in WHOLE-Donor Study

Of the 50 participants, 62% were women and 85% were Caucasian. Year of kidney donation ranged from 1985 to 2015, with a median (IQR) of 9 [6–14] years since donation at the time of interview for this study. Median (IQR) age at donation was 46.5 (36–55.5) years, with a range of 20–69 years (Table 1). At the time that these donors joined the parent study, 78% were employed and 74% were married.

### Concerns about and experiences of donation among participants and their families

We identified 31 themes regarding concerns about and experiences of donation that participants reported personally having or hearing from family and friends (Tables 2 and 3). Of these, 19 were identified as concerns, and 96% of participants reported at least one of these concerns.

**Table 2:** Proportion of participants who reported having had pre-donation, post-donation, or family concerns, by demographic characteristics

	Pre-donation		Post-donation		Family or friends	
Characteristic	%	p	%	p	%	p
Sex						
Female	81	> 0.9	65	0.8	77	0.1
Male	79		58		53	
Race						
Caucasian	83	0.4	59	0.5	76	0.02
Other race	67		78		33	
Employment status^a^						
Unemployed	79	> 0.9	50	0.3	64	0.7
Employed	81		67		69	
Marital status^a^						
Unmarried	69	0.3	81	0.07	88	0.055
Married	85		53		59	

^a^Employment and marital status at time of survey 1 administration in WHOLE-Donor Study

Significantly more Caucasian participants reported hearing concerns from family or friends than participants of other races

When asked about pre-donation concerns, 20% of participants reported no concerns, 44% reported one concern, and 36% reported two or more concerns. After donation, 38% of participants reported no concerns, 46% reported one concern, and 24% reported two or more concerns. From family and friends, 32% of participants reported hearing no concerns, 44% heard one concern, and 24% heard two or more concerns.

The proportion of participants reporting any concerns pre-donation, post-donation, or from family was similar among donors regardless of sex, employment status, and marital status (Table 2). Caucasian participants were significantly more likely to report hearing at least one concern from family or friends compared to participants of other races (76% vs. 33%, *p* = 0.02). However, there were no differences in the proportion of participants who reported concerns pre-donation or post-donation by race (Table 2).

Participants reported different informational requirements before donation. Thirty-six percent stated that they were going to donate regardless of the risks, even before receiving education about donation (Table 4, Q21). Conversely, 36% of participants reported that learning more about donation helped them to overcome donation-related fears or concerns (Table 4, Q22).

### Taxonomy of donation concerns

The top concerns that participants reported having had before donation were kidney failure (22%), general long-term risks (14%), surgical risks (14%), recipient wellbeing (12%), recovery from surgery (12%), and effects on their longevity (10%). After donation, participants’ top concerns were the recovery process (24%), lifestyle limitations (12%), potential failure of their remaining kidney (12%), general long-term risks (10%), and recipient’s wellbeing (8%). Participants reported that their families and friends were most concerned about the surgical risks (26%) and general long-term risks (22%) and 16% reported that family and friends perceived that the risks of donation endangered the donor’s family unit (Table 5, Q8).

**Table 3:** Differences in frequency of concerns that participants reported having had before donation, after donation, and heard from family or friends

Concern	Pre- donation	Post-donation	Family/Friends	p
General long-term effects	14%	10%	22%	0.3
Recovery process	12%	24%	2%	< 0.01
Recipient’s health	12%	8%	0%	0.04
Surgical risk	14%	0%	26%	< 0.01
LKD’s remaining kidney failing	22%	12%	2%	< 0.01
Being a related/directed donor	2%	0%	0%	> 0.9
Lifestyle limitations	2%	12%	2%	0.051
Endangering the family unit	0%	0%	16%	< 0.01
Not allowed to be donor	8%	0%	0%	0.03
Incision or scar	2%	4%	0%	0.8
Spouse being afraid^a^	3%	0%	15%	0.02
Future family need for a donated kidney	6%	0%	4%	0.4
Donated kidney would fail	8%	4%	0%	0.2
Insurance issues	4%	0%	2%	0.8
Effect on longevity	10%	0%	0%	0.01
Mental health	0%	2%	0%	> 0.9
Future pregnancies^b^	13%	0%	0%	0.3
Religious concerns	0%	0%	2%	> 0.9
Employment	4%	2%	0%	0.8

^a^These percentages were calculated among participants who were married at the time of donation (*N* = 34) because of the specific relevance of “spousal fear” to this population

^b^These percentages were calculated among participants who were female and < =50 at the time of donation (*N* = 15) because of the specific relevance of this concern to women of child-bearing age

**Table 4:** Other living kidney donation experiences that participants reported

Experience	%	Representative Quote
Having others more worried than donor about donation	44%	Q20: “I think my family was much more afraid than I was.”
Having blind faith in the donation process	36%	Q21: “It just hit me in the head, like, why not? Let’s see if it will work. I never had any fear. I had total faith in it. I don’t know why.”
Having more knowledge helps	36%	Q22: “I just tried to get fully educated on it, as did my family… It lessened all the concerns a lot to the point where there wasn’t a lot of concern going into it.”
Quality of care	32%	Q23: “It’s my honor to have gone where I have gone, and Johns Hopkins was absolutely wonderful.”
Sense of helping others	26%	Q24: “How relatively easy it is to do something that has an incredible benefit for somebody else. I wish I had known that.”
Being a non-directed donor	14%	Q25: “I would have liked more counseling about the ‘what ifs’ if I did decide to know the recipient.”
Thoroughness of evaluation	12%	Q26: “I was really surprised at how thoroughly I had to be checked and rechecked and examined and re-examined.”
Donor follow-up	8%	Q27: “After you make the donation of your kidney, nobody from that hospital where you donated said, ‘Hey, come back in here so we can check you’re doing okay.’“
Pressure to donate	6%	Q28: “In some ways I feel like related donors need even more protection because of this underlying assumption that you’ll do it and it leaves very little space to say you don’t want to do it.”
Cost or finances^a^	4%	Q29: “We qualified for one of those grants because we’re a couple. Maybe have information on there about the grants.”
Donor entitlement	2%	Q30: “There was a sense of entitlement from some recipients, their physicians, their teams. There was a thought that you’re like taking a medication off the shelf.”
Race	2%	Q31: “Some people, being African American, were like, I’m not going to give up a body part to anyone.”

^a^In the context of this study, associated costs were only mentioned in a positive context, such as receiving financial support

**Table 5:** Concerns about living kidney donation that participants reported having personally or hearing from family or friends

Concern	Representative quote
General long-term effects	Q1: “I knew it was a fairly routine surgery, but how would it affect me not in 2 years, 3 years, but in 10 years, 20 years.”
Recovery process	Q2: “I felt the operation would be a huge deal. Painful recovery, long recovery, the whole thing.”
Recipient’s health	Q3: “I didn’t have any concerns when I considered giving. My concern was for my recipient.”
Surgical risk	Q4: “Understanding the risks in general of having a surgery, especially one that I wasn’t supposed to get any medical benefit from.”
Donor’s remaining kidney failing	Q5: “The biggest concern was what would happen as I got older, and knowing that kidney function can sometimes decline as you got older. Would that potentially put me at risk for kidney failure?”
Being a related or directed donor	Q6: “I had another brother who was showing signs of microscopic hematuria, so I was afraid this was something familial for us.”
Lifestyle limitations after donation	Q7: “You know, things that I shouldn’t do or things that I would need to avoid, medications that I would need to avoid.”
Endangering the family unit by donating	Q8: “I think it was just a concern that I would jeopardize my own health and therefore my long-term ability to provide for my own family.”
Not allowed to be donor	Q9: “My primary concern was that… something would happen and I wouldn’t be acceptable for some reason.”
Incision or scar	Q10: “That was one of the biggest things I was worried about – what the scars would end up looking like.”
Spouse being afraid	Q11: “The main concern was interacting and communicating with my wife about it. She was cautious and fearful about me making that decision.”
Future family need for a donated kidney	Q12: “Will anyone else in my family need a kidney?”
Donated kidney would fail	Q13: “What happened if the kidney did not work after being placed in the recipient?”
Insurance issues	Q14: “My wife was worried about life insurance policies, and if – and even my health insurance policy.”
Effect on longevity	Q15: “Basically, would it shorten my life expectancy or anything like that.”
Mental health	Q16: “My husband was very concerned about my wellbeing, my psych health.”
Future pregnancies	Q17: “Will I have any problems with pregnancy in the future?”
Religious concerns	Q18: “As I found out after listening to them, Jews are supposed to be buried entirely, with all their organs and everything…so most of my friends were concerned about me in the coming world, in the afterlife.”
Employment	Q19: “My main concern was the length of time I would have to be off work.”

**Table 6:** Informational Resources Used

Resource	% Who Used Resource	Median (IQR) Usefulness^a^
Books	14%	5 (2,5)
Scientific journals	28%	8 (6,8)
Newspapers or magazines	27%	6 (5,8)
Websites	79% of donors since 2000^b^	8 (7,9)
Healthcare providers	100%	10 (9,10)
Other	44%	

^a^Usefulness was rated only by LKDs who reported using the resource. Usefulness was rated on a scale of 0–10, where 0 represented “not useful” and 10 represented “extremely useful”

^b^Restricted to donors since 2000 because that is when at least 50% of American adults used the internet [29]

**Table 7:** Themes related to how participants gathered information

Theme	%	Representative quote
Other living kidney donors	46%	Q32: “What I thought was nice and helpful… was talking to other live donors. That would have been helpful for me to have talked to them prior to my kidney donation.”
Reliability or accuracy of information	32%	Q33: “I didn’t want to clutter my brain with information that I wasn’t sure was correct.”
Availability of information/resource	16%	Q34: “I certainly counted a lot more on the information that [healthcare providers] provided, but I didn’t have access to them all the times that I wanted.”
Understandable information	14%	Q35: “Ease of understanding. Sometimes it’s too much medical terminology and mumbo jumbo, so if it’s explained in everyday language, it’s very helpful.”
Organization of information	2%	Q36: “The resources that I appreciated the most… were well organized so I could find the answers to specific questions.”
Advertisement	2%	Q37: “That it was not an advertisement or something like that.”

**Table 8:** Importance of qualities in an informational resource

Quality of resource or information	Median (IQR) Importance^a^
“It came from a reputable source.”	10 (10,10)
“It was easy to read and understand.”	10 (9,10)
“It had the information I was looking for.”	10 (8,10)
“It came recommended by a friend.”	5 (3,7)
“It came recommended by a healthcare provider.”	10 (9.5,10)
“It was easy to find.”	10 (7,10)

^a^Scale of 0–10, where 0 represented “not important” and 10 represented “extremely important”

Participants reported personally having the following concerns more often than they reported hearing them from family or friends: failure of the donor’s remaining kidney (32% vs. 2%, *p* < 0.01), the recovery process (28% vs. 2%, *p* < 0.01), the recipient’s well-being (20% vs. 0%, *p* = 0.03), and failure of the donated kidney (12% vs. 0%, *p* = 0.03). In contrast, participants reported hearing about endangering their family unit more often from friends and family than worrying about it personally (8% vs. 0%, *p* < 0.01).

Among participants, 44% reported having been less concerned overall than their family or friends about donation (Table 4, Q20). Having a spouse express fear about the donation was reported by 15% of participants who were married at the time of donation (Table 5, Q11); interestingly, one spouse did not express these fears until just before the donation. One participant who felt that the concerns of family and friends came from a lack of knowledge suggested that transplant centers create a “Frequently Asked Questions” page that donors could distribute to their social networks to facilitate discussions about donation.

### Information participants desired prior to donating

When asked if there was anything they wished they had known prior to donation, 26% of participants gave a positive response. These participants wished they had had more information about a specific complication they later experienced (38%), a better understanding of differences in transplant centers’ protocols (16%), more knowledge of how easy the donation process would be (8%), and more practical information regarding the recovery process (38%). This practical information was framed as advice for future living kidney donors, including the importance of walking as soon as possible after surgery, despite the initial discomfort; requesting anti-nausea medication after surgery; knowing that the car ride home can be very painful; knowing what clothes are most comfortable after surgery; and understanding that it takes a different amount of time for each donor to feel “normal” after surgery. Of the participants who, in hindsight, desired more information pre-donation on a specific topic, none indicated that having this information before surgery would have prevented them from donating, although they were not explicitly asked about that topic.

### Participants’ information-gathering behaviors

Participants reported getting information from health care providers (100%), websites (79% of donors since 2000), scientific journals (28%), newspapers or magazines (27%), books (14%), and other resources (44%) including other donors, pamphlets, and personal networks (Table 6). The resources with the highest median (IQR) ratings for usefulness were for healthcare providers [10 (9, 10)], websites [8 (7,9)], and scientific journals [8 (6,9)] (Table 6).

When learning about donation, 26% of participants used other living kidney donors as a source of information. Of these donors, 46% had a family or friend who had previously donated, 38% learned about other donors’ experiences on blogs, forums, and YouTube, and 15% did not specify how they had communicated with other donors. Among participants who had pre-donation contact with other living kidney donors, 46% reported that this contact made them more confident in their own decision to donate. As one participant stated, “There were some concerns voiced but not enough to stop me… Plus, my sister had donated a kidney to my brother… 10 years earlier… I had a living example of a donor.” Since their own donations, 6% of participants had served as a resource to other potential living kidney donors. During the interviews, 4% made unsolicited offers to speak to future living kidney donor candidates at our center because they thought that access to a prior donor was so important.

In free-response questions, participants reported that, when gathering information about donation, they considered the source’s reliability or accuracy (32%), availability (16%), clarity (7%), and organization (2%). Among participants, 2% reported avoiding resources that were advertisements (Table 7, Q37). When asked to rate the importance of specific qualities of an informational resource from 0 to 10, the reported median (IQR) importance of the following characteristics was highest for: the reputability of its source [10 (10,10)], its clarity [10 (9,10)], its content being what the participant sought [10 (8,10)], its recommendation by a healthcare provider [10 (9.5,10)], and whether it was easily found [10 (7,10)] (Table 8). The recommendation of a resource by a friend was, overall, rated as less important [median (IQR) 5 (3,7)], although 8% of participants rated its importance as a 10. Of those who rated the importance of a friend’s recommendation as a 10, one stated that his friend was a nurse and worked in organ transplantation, while another qualified his response by adding, “especially if it was someone who had gone through [living donation].”

## Discussion

In this single-center study of donation-related concerns, information-gathering practices, and donation experiences, we found statistically significant differences in the concerns about donation held by donors versus their family and friends. Participants reported personally having specific concerns, including the recovery process, kidney failure, ineligibility to donate, and effect of donation on longevity. On the other hand, participants reported hearing more general concerns from family and friends, such as an overall fear of complications. None of the reported concerns were associated with participant sex, race, employment status, or marital status. When gathering information about donation, participants used multiple sources including healthcare providers (100%) and the internet (79% of donors since 2000). Hearing the experiences of other living kidney donors, whether in person or through a website, was a valuable resource to 26% of participants, with an additional 20% participants reporting that they desired contact with other living kidney donors.

Participants provided heterogenous reports of how much information they required to be comfortable with donation. While 36% expressed a sort of “blind faith” in donation, stating that they knew they were going to donate despite lacking knowledge about the associated risks, an equal proportion reported that acquiring greater knowledge about donation helped allay their fears. This type of decision-making has previously been described by Hiller et al. as “moral or straightforward” decision-making [17]. Hiller et al. found that 25% of the donor population decided to donate immediately (prior to receiving any education). This study was conducted at our center, suggesting that the type of decision-making used by donors might have changed over the past two decades. Whether this is related to the change in the donor population during this time period remains unknown [22]. However, in both our study and Hiller et al.’s study, the majority of participants sought more information before making a decision to donate [17]. Increased knowledge about donation has been found to increased comfort with donation prior studies [16, 23], as well, underscoring the importance of improving educational materials and making them accessible to potential donors.

The types of concerns that were reported by donors in this study are similar to those previously noted in the literature, including length of hospital stay, out-of-pocket expenses, appearance of the surgical scar, risk of end-stage renal disease for the donor, and recipient wellbeing [14]. Donor concerns that had been reported by previous studies but that were not found in this study included time to get to the transplant center; of note, this concern was found in a study by Boulware et al. of households in Maryland [14] and people who were less concerned about travel time might have been more likely to end up donating. Donors in our study reported that health care providers were a main source of information, consistent with the findings of Waterman et al. [18]. However, Waterman et al. found that reading brochures was the other most common source of information, while donors in our study used websites as their second most common source of information. This might reflect an ongoing trend towards patients finding medical information online and should be monitored by providers so that information is being provided to patients in the forms that are most accessible and desired.

Since participants’ social networks were a source of donation-related concerns, future educational interventions may need to target not only potential participants but also their families and friends. Prior studies have suggested that increasing education for LKDs’ social networks might allow donors’ families and friends to provide better support for donors and might increase donation rates overall [24]. While both national organizations and transplant centers have worked to develop internet resources [25–27], including resources specifically for potential donors’ social networks [28], the living donation rate has continued to decline [22]. Interviews of donors’ social networks could deepen our understanding of their concerns and misconceptions and guide the creation of targeted resources for donors’ support systems.

Our sample population was of limited size and drawn from a single center, but the purpose of the study was to perform in-depth interviews and capture themes that would go unmeasured by more quantitative analytical tools. Although our study is limited by its retrospective nature, misreporting and memory biases in retrospective studies of LKD experiences have been found to be minimal [24]. Our use of a convenience sample could have introduced a selection bias, but we found no significant demographic differences between the participants and donors who were contacted but did not participate in our study. For quantitative analysis, the sample size of 50 participants might limit our ability to detect differences in concerns by participant demographics. However, the distribution of concerns was comparable across demographic subgroups and we were able to detect statistically significant differences between the concerns LKDs experienced personally and those they heard from family and friends. Furthermore, we continued data collection past thematic saturation, which should have enabled us to capture all major themes.

## Conclusions

In summary, nearly all participants reported personal or family concerns regarding donation. Concerns from participants’ social networks were common and often differed from participants’ personal concerns. The educational efforts of the transplant community have been largely focused on potential donors – leaving these individuals to answer myriad questions from family and friends. If transplant centers are to effectively support potential donors, outreach and education must extend to caregivers, families, and friends.

## Abbreviation

LKD: Living kidney donor
